# Effects of a short individually tailored counselling session for HIV prevention in gay and bisexual men receiving Hepatitis B vaccination

**DOI:** 10.1186/1471-2458-9-255

**Published:** 2009-07-21

**Authors:** Mireille EG Wolfers, John BF de Wit, Harm J Hospers, Jan H Richardus, Onno de Zwart

**Affiliations:** 1 Division of Infectious Diseases Control, Municipal Public Health Service Rotterdam Area, the Netherlands; 2 Department of Public Health, Erasmus MC, University Medical Centre Rotterdam, Rotterdam, the Netherlands; 3 Department of Social and Organizational Psychology, Utrecht University, Utrecht, the Netherlands; 4 Department of Experimental Psychology, Faculty of Psychology, Maastricht University, Maastricht, the Netherlands

## Abstract

**Background:**

There is currently a trend towards unsafe unprotected anal intercourse (UAI) among men who have sex with men. We evaluated a short individual counselling session on reducing UAI among gay and bisexual men.

**Methods:**

A quasi-experimental design was used to evaluate the counselling session. This session was conducted during consulting hours at four municipal health clinics during a Hepatitis B vaccination campaign. These clinics offered free vaccination to high-risk groups, such as gay and bisexual men.

All gay and bisexual men attending health clinics in four cities in the Netherlands were asked to participate. Each participant in the intervention group received a fifteen-minute individual counselling based on the Theory of Planned Behaviour and Motivational Interviewing. Changes in UAI were measured over a 5-months period, using self-administered questionnaires. UAI was measured separately for receptive and insertive intercourse in steady and casual partners. These measures were combined in an index-score (range 0–8).

**Results:**

While UAI in the counselling group remained stable, it increased in the controls by 66% from 0.41 to 0.68. The results show that the intervention had a protective effect on sexual behaviour with steady partners. Intervention effects were strongest within steady relationships, especially for men whose steady-relationship status changed during the study. The intervention was well accepted among the target group.

**Conclusion:**

The fifteen-minute individually tailored counselling session was not only well accepted but also had a protective effect on risk behaviour after a follow-up of six months.

## Background

In the Netherlands, as in many industrialized counties, men who have sex with men (MSM) are at highest risk for HIV infection. Since the late 1990s, incidence of HIV among MSM remains high and increasing numbers of newly diagnosed HIV infections in MSM have been observed in western industrialised countries [1-6]. HIV-prevalence among MSM in the Netherlands is estimated at 5.3% in 2005 [7], and incidence has increased from 314 new infections in 1998 to 513 in 2006 [8]. In the past decade epidemics of STIs occurred simultaneously among MSM, suggesting an increase in sexual risk behaviour [9,10].

Among MSM, unprotected anal intercourse (UAI) is the major route of HIV transmission and the use of condoms is related to the relational status men have with their sexual partner. Studies have shown that UAI among MSM is more likely with steady partners than with casual partners, while new HIV infections are also more likely to occur within steady relationships than in casual contacts [11-13]. Many MSM in steady relationships dispense of using condoms within their steady relationship only after mutually negative HIV-testing and conditional on having made agreements about sex outside the relationship, a risk reduction strategy referred to as "negotiated safety" [14]. While a majority of MSM in steady relationships may have made sexual agreements to avoid the introduction of HIV into their couple [11,15], these agreements are not always kept [12]. Also, UAI is often initiated early in new relationships, frequently without any explicit discussion [16,17]. Furthermore, life expectancy of people living with HIV in high income countries has improved dramatically since the introduction of effective antiretroviral therapy (ART), and research among MSM in the Netherlands has shown that a decreased perception of HIV/AIDS threat not only prospectively predicts an increase in UAI, but is also related to STI incidence [18,19]

Behavioural and epidemiological data suggest a continued need to address risky sexual behaviour and to promote safe sex in the context of the type of sexual relationships in which it occurs. Not only does the prevalence of risk and protective behaviours differ reliably between steady relationships and casual sexual contacts, the appraisal of the risk of HIV transmission also differs, as do other psycho-social factors that shape condom use [12]. In the Netherlands, HIV prevention activities are provided by in a range of community and health care settings, including during HIV/STI testing and counselling, telephone hotlines, peer outreach, educational materials and Internet portals and websites. Individualized prevention that occurs as part of STI/HIV testing and counselling can in particular address the personal psychological, interpersonal and social aspects of sexual risk behaviour that are important in successful HIV prevention for MSM [20]. Although to date there is a paucity of studies evaluating the efficacy of HIV prevention through individual counselling [21,22], several studies have demonstrated that brief counselling can be effective in promoting safe sex [23-25]. The introduction of a Hepatitis B vaccination campaign for MSM in the Netherlands created a unique opportunity to also offer MSM an individual counselling session on HIV prevention, which was provided by a skilled health professional as part of men's series of consultation for HBV vaccination. The objectives of this study are to evaluate the effect of this individual HIV prevention counselling and to assess its acceptability.

## Methods

A quasi-experimental study using a pre-test post-test design was conducted in four Municipal Public Health Services (MPHS) in the Netherlands during a national campaign for free Hepatitis B vaccination among MSM. Between June 2003 and January 2005 every gay and bisexual male visiting one of the participating MPHSs for the purpose of a vaccination against Hepatitis B Virus (HBV) was asked to participate in the study. To be eligible for free HBV vaccination individuals had to identify as members of a population group at increased risk for HBV infection, including MSM; this information was obtained at intake. At one of the participating four MPHSs, in Rotterdam, the second largest city in the Netherlands, respondents received a 15-minute individual HIV prevention counselling during the visit for their second vaccination. MSM who obtained HBV vaccination at the three other participating MPHSs, which together serviced two other major urban regions in the Netherlands (Utrecht and Groningen, the fourth and eighth largest cities In the Netherlands, respectively) participated as controls, receiving standard care with the HBV-vaccination.

The HIV prevention counselling was delivered by public health nurses who used a protocol that was specifically developed for this purpose. The aim was to reduce UAI with serodiscordant or unknown sex partners. Using the Theory of Planned Behaviour [26], relevant determinants of condom use intentions with casual and steady partners were addressed. Based on previous research among Dutch MSM, self-efficacy was considered a key factor shaping condom use in casual relationships, while partner norms were considered most influential in shaping men's use of condoms within their steady relationships [11]). Elements of Motivational Interviewing [27], including such techniques as using empathy, creating cognitive dissonance, responding to resistance and eliciting change talk, were used to motivate participants to change their sexual risk behaviour. The counselling protocol was tailored to men's actual relational status (steady partner only, casual partners only, or steady partner and concurrent casual partners) and to current sexual and risk practices.

Participants received no payment or other incentive for participation and a declaration of no objection for this study was given by the medical ethical committee of the Erasmus University Medical Center, Rotterdam.

### Evaluation design

Participants in the experimental and control groups completed pre-test assessments after obtaining their first HBV vaccination (month 0); post-test assessments where obtained after men received their third and final vaccination (month 6). Men in the experimental condition received individual HIV prevention counselling after obtaining their second HBV vaccination (month 1). Informed consent was obtained at entry into the study, after which participants completed the pre-test paper-and-pen questionnaire that assessed demographic characteristics, HIV-test history, sexual behaviours and condom use in the previous six months. The post-test questionnaire equally assessed sexual behaviours and condom use in the previous six months. At post test men in the experimental group also rated their appreciation for the intervention. In the experimental group, the average time between receiving HIV prevention counselling and completing the follow-up questionnaire was 5.6 months.

### Outcome measures

The outcome measure was self-reported condom use in the previous six months for receptive and insertive anal intercourse with casual and steady partners. Condom use was measured on a 5-point Likert scale, with answers ranging from never to always using condoms. A comprehensive risk behaviour measure (UAI-index) was constructed that combined the four specific sexual risk behaviours that were assessed, i.e. receptive and insertive UAI with steady and casual partners (range 0–8; see below). In addition, separate measures for steady and casual partners were computed, combining receptive and insertive UAI (range 0–4). For each sexual practice not engaging in the specific form of anal intercourse or using condoms always/most of the times was coded 0 (lowest risk), sometimes using a condom was coded 1 (moderate risk), and mostly not/never using a condom was coded 2 (highest risk). Risk reduction in steady relationships was taken into account by attributing the value 0 when men engaged in UAI with a steady partner who had equally tested HIV-negative, and when they had made agreements about safe sex outside the relationship.

### Appreciation of the intervention

On four items participants' general appreciation of the counselling was rated, measuring on 5-point Likert scales, answers ranging from very pleasant to very unpleasant; very useful to very useless; very nice to very boring; and very informative to not at all informative, Crohnbachs alpha = 0.85. Another three single items assessed participants' opinion regarding personal relevance and personal experience with the counselling, using 5-point Likert scales for agreeing to disagreeing ("Did you think the topics discussed were personally relevant to you?"; "Did you speak frankly about personal matters related to HIV and sexuality?" and: "How burdensome did you think the counselling was to you?").

### Statistical analyses

Analyses were conducted using SPSS 15.0. To compare demographic characteristics of experimental and control groups, chi-square test, student's t-test and analyses of variance were used, depending on the measurement level of the data. To determine intervention effects on outcome measures, change scores were computed between the UAI index-scores on pre-test and post-test. Differences in risk behaviour change between experimental and control groups were then tested by analyses of covariance (ANCOVA), adjusted for educational level (higher versus lower education) and ethnic background (Dutch versus non-Dutch). To estimate whether changes in outcome measures could be contributed to a regression to the mean, models were re-run while adding the UAI-index score at pre-test as covariate, a method that is used to adjust observed changes for regression to the mean effects [28,29].

## Results

### Participants

**Figure 1:**
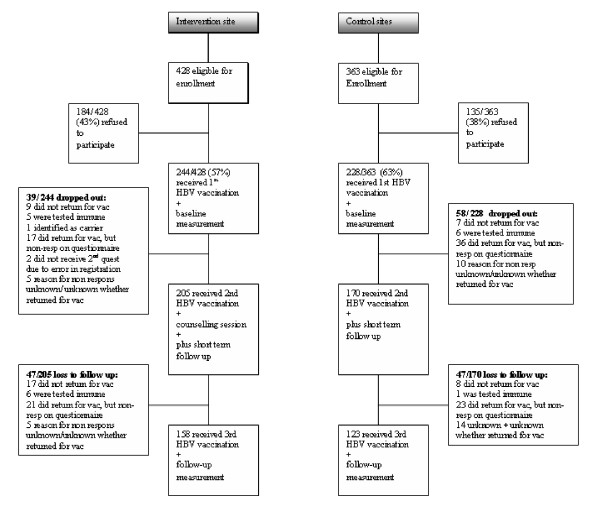


During the inclusion period, 428 MSM were eligible for enrolment at the experimental site; 363 MSM were eligible at the control sites (see Figure 1). A total of 244 men (57%) at the intervention site and 228 (63%) men at the control sites consented to participate at the time of their first vaccination, and completed the pre-test assessment. After one month, 205/428 (48%) men returned for their second vaccination at the intervention site and received the individual HIV counselling; complete follow-up was achieved for 158/205 (77%) of these men. At the control sites, 170/363 (47%) returned for their second vaccination, and complete follow-up was achieved for 123/170 (72%) of these men.

Table 1 presents a comparison of the demographic and behavioural characteristics of MSM in the experimental and control groups; only participants who completed pre-test and post-test assessments are included in this comparison. The average age of the study sample was 36.6 years (SD = 11.0). Although participants primarily had a Dutch ethnic background, more men in the experimental group had a non-Dutch ethnic background compared to controls (13.5% versus 5.0%; χ^2 ^= 5.681, p < 0.05). At pre-test, relationship status, numbers of partners in the preceding six months and the proportion of men practicing UAI were comparable between the experimental and control groups. Most men (47%) reported casual sex partners only and one third reported steady as well as casual partners. Thirteen percent of the men reported steady relationships only, and 8% reported no partners. Eighty-seven percent of the participants (n = 71) who reported anal sex with steady partners in the preceding six months reported UAI with these partners. Leaving out the men who made agreements about safe sex after mutually negative HIV-testing, the proportion of men engaging in risky anal sex with their steady partner decreased to 43%. Eighteen percent (n = 41) of participants who had anal sex with casual partners in the past six months reported UAI with these partners. History of testing for STI or HIV was also comparable between the groups: 62% had previously tested for HIV and 37% had tested for other STIs; 3% reported to be HIV-positive and 21% ever tested positive for STI.

**Table 1:** Characteristics of participants at pre-test who completed all study visits (n = 281)

	Total (n = 281)		Experimental group (n = 158)		Control group (n = 123)		Difference between study conditions	
	No.	%	No.	%	No.	%	χ^2^	p

Education^1^								
Low	93	33.1	59	37.3	34	27.6		
High	187	66.5	98	62.0	89	72.4	3.07	0.08
								
Ethnicity								
non-Dutch	27	9.6	21	13.3	6	4.9		
Dutch	249	88.6	134	84.8	115	93.5	5.68	0.02
								
Sexual orientation								
men only	259	92.2	146	92.4	113	91.9		
men and women	12	4.3	7	4.4	5	4.1	0.02	0.89
								
Ever tested for STI^2^	104	37.0	62	39.2	42	34.1	0.83	0.36
Tested positive for STI^2^	22	21.2	10	16.1	12	30.8	2.72	0.10
Tested for HIV	175	62.3	97	61.4	78	63.4	0.19	0.66
Tested HIV positive	6	3.4	3	3.1	3	3.8	0.08	0.96
								
Relationship status^2^							3.46	0.33
no partners	21	7.5	13	8.2	8	6.5		
steady partner	36	12.8	22	13.9	14	11.4		
steady partner and casual partners	91	32.4	44	27.8	47	38.2		
casual partners	133	47.3	79	50	54	43.9		
								
Anal sex with steady partner	91/127	71.7	47/66	71.2	44/61	72.1	0.01	0.92
Anal sex with casual partner	224/224	100	123/123	100	101/101	100	0.78	0.38
Unprotected Anal Intercourse (UAI)^2^								
with steady partner^3^	71/91	78.0	38/47	80.9	33/44	75.0	0.51	0.78
with steady partner (mutual HIV negative serostatus^4^)	39/91	42.9	19/47	40.4	20/44	45.5	1.04	0.31
with casual partners^5^	41/224	18.3	24/123	19.5	17/101	16.8	0.27	0.61
								
	Mean	SD	Mean	SD	Mean	SD	t	p
Age	36.6	(11.04)	36.2	(11.25)	37.0	(10.8)	-0.55	0.58
Number of partners^2,6^	4.9	(5.84)	4.8	(6.0)	5.1	(5.64)	-0.36	0.72

^1 ^Low: elementary school, vocational school or lower-level high school; high: university, college, or higher- level high school

^2 ^in past six months

^3 ^percentage of respondents who had anal sex with steady partner

^4 ^and agreements about safe sex outside the relationship

^5 ^percentage of respondents who had anal sex with casual partner

^6 ^16 respondents in the experimental group and 7 respondents in the control group had casual partners but did not report number of casual partners.

One reason for participant drop-out was non-compliance with the second and third HBV-vaccination. Of the 472 participants who completed the pre-test assessment, 41 (9%) did not return for their second or third vaccination. Furthermore, as a result of the blood-test after the first vaccination, 19 men (4%) were excluded from the study since no further vaccination was given (18 men tested immune for HBV and one man was identified as an HBV-carrier). Ninety-seven participants (20%) did present for the third vaccination but did not fill in the post-test questionnaire; two men did not receive the post-test questionnaire due to an error in the registration system. For 34 participants (7%) it was unknown whether they returned for vaccination. In the experimental group, men lost to follow-up reported more unsafe sexual behaviour at pre-test than those retained (UAI score 0.87 versus 0.42; t = 2.7, p < 0.05). In the control group, no significant difference in unsafe sexual behaviour was found between men who were lost to follow-up and those who were retained. We also compared baseline characteristics between men who were retained or lost to follow-up. In the experimental group, those men who were retained were higher educated (62% versus 44%; χ^2 ^= 7.50, p < 0.01) and more often had a Dutch ethnic background than those who were lost to follow-up (87% vs 72%; χ^2 ^= 7.51, p < 0.01). In the control group no significant differences in educational level and ethnic background were found between men who were lost to follow-up or retained.

### Effect of the intervention

#### Risk-taking with steady and casual partners

At pre-test, men on average scored 0.41 on the overall UAI-index (insertive and receptive anal sex with steady and casual partners combined, see Table 2). A series of ANCOVA's was performed to test difference in UAI change scores between the intervention and control condition, with ethnicity and educational level entered as covariates (Model 1). Table 2 shows the results of these analyses, for insertive and receptive anal sex with steady and casual partners combined, and for steady and casual partners separately. The analysis of the overall UAI change score produced non-significant effects for the covariates; ethnic background, F(1,266) = 0.05, and educational level, F(1,266) = 0.02. Importantly, a significant difference in UAI change score was found between the experimental group and the control group. While the UAI-index score remained stable in the experimental group, it had increased by 0.27 in the control group, F(1,266) = 4.85 p < 0.05. Adding the baseline UAI-index score as a further covariate to control for potential regression to the mean effects (Model 2), also resulted in a significant difference between the experimental and control groups, implying that a regression to the mean effect is unlikely to explain the observed difference in change. Differences between experimental and control groups in UAI change score for steady and casual partners separately were not statistically significant.

**Table 2:** A comparison of unprotected anal intercourse (UAI)-change scores (t2-t0) between experimental and control group at follow up.

		Experimental group			Control group		Model 1		Model 2	
	n	Pre-test UAI-score	UAI-change score	n	Pre-test UAI-score	UAI-change score	F	p	F	P

UAI with casual and steady partners	152	0.42	-0.05	118	0.41	0.27	4.85	<0.05	5.72	<0.05
UAI with steady partners	152	0.25	0.04	118	0.33	0.27	2.48	0.12	3.90	<0.05
UAI with casual partners	155^1^	0.17	-0.08	121^1^	0.08	0.00	1.94	0.17	0.27	0.61

^1^The number of respondents for the different behaviours are not equal, due to more missing values in the variables used to calculate the UAI-index score for UAI with steady partners than with casual partners

Note:

Model 1: covariates include ethnic background (Dutch vs non-Dutch) and educational level (low level: elementary school, vocational school or lower-level high school vs high level: university, college, or higher- level high school)

Model 2: As Model 1, with pre-test UAI- score as additional covariate

#### Impact of change in steady-relationship status

During the study period, 18% of participating men changed their steady-relationship status. These men either became newly involved in a steady relationship; lost the steady partner they had at pre-test without entering a new steady relationship; or lost their steady partner and started a new steady relationship. Steady-relationship status remained unchanged for 82% of the participants; these men reported having the same steady partner or consistently had no steady partner. A change in steady-relationship status can affect the UAI-index score, as can the extent to which men engaged in UAI at pre-test. To provide a more controlled comparison of differences in changes in UAI-index scores between the experimental and control groups we conducted a three-way analysis of variance with intervention-condition (experimental vs control), steady-relationship change (no vs yes) and sexual behaviour at pre-test (safe vs unsafe) as independent factors. In table 3, 4 and 5 means of UAI-index scores are presented for the overall score and the scores for steady and casual partners separately. Additional file 1 gives test results for the series of ANCOVA's that was performed.

**Table 3:** UAI with steady and casual partners.

		Experimental group			Control group		
SR^2 ^status change	behaviour at pre-test	n	Mean	SD	n	Mean	SD

no	Unsafe	26	-0,58	1,27	31	0,00	1,32
	Safe	94	0,10	0,51	66	0,20	0,77
	Total	120	-0,05	0,78	97	0,13	0,97
yes	Unsafe**	13	-0,92	2,43	4	0,50	2,52
	Safe	16	0,69	1,08	13	1,46	1,94
	Total**	29	-0,03	1,95	17	1,24	2,05
Total	Unsafe**	39	-0,69	1,72	35	0,06	1,45
	Safe	110	0,18	0,65	79	0,42	1,14
	Total**	149	-0,05	1,10	114	0,30	1,25

Means and standard deviations (SD) for Unprotected Anal Intercourse (UAI)-change score (t2-t0) for insertive and receptive anal sex by study condition, steady relationship(SR)-status change (yes vs no) and sexual behaviour at pretest (safe vs unsafe)^1^.

^1 ^ANCOVA adjusted for ethnic background (Dutch vs non-Dutch) and educational level (low level: elementary school, vocational school or lower-level high school; high level: university, college, or higher- level high school).

^2 ^SR = steady relationship

** significant difference between experimental group and control group, P < 0.01

**Table 4:** UAI with steady partners.

		Experimental group			Control group		
SR^2 ^status change	behaviour at pre-test	n	Mean	SD	n	Mean	SD

no	unsafe	9	-0,44	1,33	17	-0,18	1,51
	safe	111	0,11	0,59	81	0,17	0,72
	Total	120	0,07	0,68	98	0,11	0,91
yes	Unsafe**	9	-1,89	2,26	2	-1,00	1,41
	Safe*	20	0,75	1,41	15	1,53	1,96
	Total**	29	-0,07	2,09	17	1,24	2,05
Total	unsafe	18	-1,17	1,95	19	-0,26	1,48
	safe	131	0,21	0,80	96	0,39	1,12
	Total**	149	0,04	1,10	115	0,27	1,20

Means and standard deviations (SD) for Unprotected Anal Intercourse (UAI)-change score (t2 – t0) for insertive and receptive anal sex by study condition, steady relationship(SR)-status change (yes vs no) and sexual behaviour at pretest (safe vs unsafe)^1^.

^1 ^ANCOVA adjusted for ethnic background (Dutch vs non-Dutch) and educational level (low level: elementary school, vocational school or lower-level high school; high level: university, college, or higher- level high school).

^2 ^SR = steady relationship

* significant difference between experimental group and control group, P < 0.05

** significant difference between experimental group and control group, P < 0.01

**Table 5:** UAI with casual partners.

	Experimental group			Control group		
behaviour at pre-test	n	Mean	SD	n	Mean	SD

unsafe	23	-0,78	1,20	17	0,06**	1,39
safe	132	0,04	0,26	104	0,01	0,09

Total	155	-0.08	0,59	121	0,00***	0,52

^Means and standard deviations (SD) for Unprotected Anal Intercourse (UAI)-change score (t2 – t0) for insertive and receptive anal sex by study condition, steady relationship(SR)-status change (yes vs no) and sexual behaviour at pretest (safe vs unsafe)^1^.^

^1 ^ANCOVA adjusted for ethnic background (Dutch vs non-Dutch) and educational level (low level:

elementary school, vocational school or lower-level high school; high level: university, college,

or higher- level high school).

* significant difference between experimental group and control group, P < 0.05

** significant difference between experimental group and control group, P < 0.01

Table 3 presents the results of these analyses for the overall UAI-change score, combining insertive and receptive anal sex with steady and casual partners, adjusted for ethnic background and educational level. The covariates ethnic background and educational level were not significantly related to UAI-change scores.

The analysis produced significant main effects on UAI-change score for the study condition, F(1,254) = 12.58, p < 0.001, steady relationship change, F(1,254) = 6.12, p < 0.05 and sexual risk behaviour at baseline, F(1.254) = 20.27, p < 0.001. Adding the baseline UAI-index score as a covariate resulted in significant main effects again for study condition and steady relationship change, implying that a regression to the mean effect in the UAI change score is unlikely to explain these effects. The effect of sexual risk behaviour at baseline did not reach statistical significance anymore. Adjusted for pre-test UAI-index scores, a significant interaction between study condition and steady relationship change was found, F(1,254) = 4.00, p < 0.05, but neither the interaction between study condition and sexual risk behaviour at baseline, nor the three-way interaction between study condition, steady-relationship change and sexual risk behaviour at baseline reached statistical significance. Further examination of simple effects showed that there was a significant difference in UAI-change score between the experimental and control groups for the men who had a steady-relationship change, F(1,266) = 8.97, p < 0.01, but not for men whose relationship status had not changed, F(1,266) = 1.95.

Table 4 shows the separate analysis for anal sex with steady partners. The covariates ethnic background and educational level were not significantly related to UAI-change scores, while there were significant main effects on UAI-change scores of study condition, F(1,255) = 4.96, p < 0.05 and sexual risk behaviour at baseline, F(1,255) = 50.38, p < 0.001, the effect of steady-relationship change was not significant. Adding the pre-test UAI-index score resulted in a significant main effect for study condition again, implying that a regression to the mean effect is not likely to explain differences in the observed changes between the experimental and control group. After adjusting for the pre-test UAI-index score, significance was also found for the effect of steady-relationship change on UAI-change scores, F(1,255)- = 6.77, p < 0.05, but not for sexual risk behaviour at baseline. A significant interaction between study condition and steady-relationship change, and a non-significant effect between study condition and sexual risk behaviour was found. A three-way interaction between study condition, steady-relationship change and sexual risk behaviour at pre-test did not reach statistical significance. Simple effect analyses showed significant differences between the experimental and control groups for men who had changed their steady-relationship status, F(1,266) = 10.65, p < 0.001, but not for men whose steady-relationship status had not changed, F(1,266) = 0.16.

Table 5 shows the analysis for UAI with casual partners, which again did not show any significant effects for ethnic background and educational level. Significant main effects were found for study condition and risk behaviour at pre-test, but after adjusting for the pre-test UAI-index scores these effects did not remain significant, F(1,270) = 0.36, p = 0.55. This suggests that the observed difference in UAI-change score between study conditions reflects a regression to the mean effect. Also no significant interaction effect was produced between the experimental condition and the behaviour at pre-test, adjusted for pre-test UAI-index scores, suggesting that there were no changes in UAI-index score for the behaviour with casual partners within groups of men who were different in risk behaviour at pre-test, other than the natural variation.

To illustrate the changes across the four different behaviours that are used for calculating the UAI-index score, table 6 presents the scores for each behaviour in the experimental and control groups. It shows that behaviour with steady partners in the control group had changed (more UAI especially in receptive AI) and that behaviour with casual partners in the intervention group had changed, they had less insertive UAI.

**Table 6:** Descriptives of scores on unprotected anal intercourse in intervention and control group, for steady and casual partner, on pre-test and post-test

			Experimental group n = 158		Control group n = 123	
			Pre-test	Post-test	Pre-test	Post-test
		UAI-score	n	n	n	n
Steady partner	UIAI	0	134	133	105	99
		1	1	2	0	1
		2	23	23	18	23
		Av score	0,60	0,58	0,60	0,76
	URAI	0	133	132	101	90
		1	1	2	1	2
		2	24	24	21	31
		Av score	0,61	0,63	0,72	1,03
Casual partner	UIAI	0	144	152	118	117
		1	8	6	4	5
		2	6	0	1	1
		Av score	0,28	0,16	0,15	0,15
	URAI	0	153	152	120	119
		1	3	4	2	3
		2	2	2	1	1
		Av score	0,13	0,15	0,10	0,11

### Appreciation of the intervention

On four items rating participants' general appreciations of the counselling, participants who received the counselling rated it very positively with an average score of 4.0 on a scale from 1–5. Ninety-five percent reported that they had spoken frankly; 89% reported that the subjects discussed were personally relevant, and 89% considered the counselling not burdening or stressful.

## Discussion

This study evaluated the efficacy of a single individually tailored counselling session to reduce unprotected anal intercourse among MSM. The results show that the intervention had a protective effect on sexual behaviour; six months after the time of the intervention, risk behaviour of men in the experimental group remained stable while it had increased by 0.27 in the control group. The intervention seemed mainly to have affected behaviour with steady partners.

While the intervention group did not change, the controls reported more unsafe behaviour. It might be that this reflects a wider trend towards more unsafe sexual behaviour among MSM, both in the Netherlands and internationally [2,6,19,30,31]. Other authors have also reported intervention studies in which they found an increase in risky sexual behaviour in the control group [32,33]. It could also be that the HBV vaccination itself has produced unwanted side-effects. Notably, men who received the vaccination may feel protected and perceive less risks when they have unprotected anal sex. The HIV prevention counselling may have protected men in the intervention group from this side-effect. Maybe risk perception regarding UAI in a steady relationship is stronger for HBV-infection risks than for HIV-infection, as a result men feel safer after the HBV vaccination to engage in sex without condoms. In the intervention group this has not occurred due to the protective effect of the counselling-intervention.

When considering the results it is important to note that sexual risk behaviour in the study sample was rather low. Compared to a recent large cross-sectional study among Dutch MSM [12], UAI with casual partners was low: 18% of respondents who had anal sex with casual partners reported UAI with these partners; this contrast with the 41% reported by Hospers et al. Although UAI with steady partners was similar (78%), only a minority in our study group reported having a steady partner. Other studies on short counselling sessions have targeted MSM during voluntary HIV counselling and testing, these groups reported higher risk behaviours [23,34]. Despite the small numbers of participants who reported risk behaviour at baseline, it was possible to assess a positive effect of the intervention.

The present study has several limitations. A first limitation is related to the design of the study. Because of the quasi-experimental design the causality of the effect of HIV- prevention- counseling relation on behavioural change must be interpreted with caution. A lack of randomnisation may have resulted in regression to the mean effects, natural variation between pre-test and post-test may then be interpreted as a real change. However, we used analysis of covariance with the change between baseline and follow-up as the outcome variable. To adjust observed measurements for regression to the mean effects we-run the models with the scores at pre-test as covariates. We found that a regression to the mean effect was unlikely to explain the observed difference in changes in the overall UAI-index score and the score with steady partners, but that changes in UAI with casual partners are most likely explained by a regression to the mean.

Respondents in the experimental and control group were recruited in different cities in the Netherlands, but we chose cities that were all situated in major urban regions of the country. These cities have a rather comparable gay scene, with an easy accessible municipal health service situated in the city center which offered the free HBV vaccinations. We do not expect that the risk behaviour between the men living in the different cities varies substantially, as the Netherlands are not very large and men tend to travel to gay nightlife events. Also HIV prevention activities are comparable between the cities. In the Netherlands, HIV prevention aimed at risk groups, such as MSM, is carried out by the local municipal health services and is supported by a national organization on gay and lesbian health.

Secondly, the power of this study is lower then was calculated, since the recruitment of the men was less then expected. To obtain sufficient power, 216 men in each group were needed, with at least 40% risky behaviour. We were able to recruit 472 men, but the large drop out reduced this number to 281, and less than 30% of the sample reported risk behaviour. Due to the small numbers of respondents we could not differentiate between the different types of change in men's steady-relationship status. Unfortunately, it therefore remains unclear how steady relationship change is related to a change in risk behaviour. For example, men who lose their steady relationship can become safer, because they do not practice UAI with their steady partner, but they can also become more unsafe because they engage in unsafe UAI with a new partner or casual partners.

Another limitation is a high drop-out-rate after completion of the pre-test assessment: 191 of the 472 participants (40%) who initially agreed to participate did not finish the study. A major reason for this drop-out was that participants did not obtain their second or third Hepatitis B vaccination at the same research site. Men may have obtained these vaccinations at a different health service and a national on-line registration made it easy to continue or finish the vaccination programme elsewhere. Participants in the experimental group who obtained their second vaccination elsewhere did not receive the HIV prevention counselling, and participants who had the third vaccination elsewhere could not complete follow-up. The questionnaire was anonymous; participants were only asked their year of birth, the first letter of their surname and first name, and the date of vaccination. Therefore it was not possible to trace them in the on-line registration to check whether they had completed their series of vaccination elsewhere or did not comply to follow up vaccinations.

Participants could also drop out as a result of their blood test. After the first vaccination, blood was taken to test whether a person had already been in contact with the Hepatitis B virus and had acquired natural immunity against the virus or had become a carrier. The second and third vaccinations were not given to men with natural immunity or men who were chronic carriers of HBV. The drop-out was selective: respondents in the intervention group who dropped out reported more UAI at baseline than those who received the intervention and completed follow-up measurement. By attenuating potential effects, this may have resulted in an underestimation the effect of the counselling. It may, however, also have resulted in an overestimation if men with riskier behaviour are less likely to change their behaviour after a brief counselling session.

Another limitation is that we used proportion measures; we measured condom-use on 5-point Likert-scales. A drawback of using this type of scale is that individuals could liberally interpret the categories (never to always) [35]. However, we computed an index score using 4 items, specified for partner-type (casual and steady) and sex act (receptive and insertive anal intercourse). This multi-item measure gives a broader assessment of safe sexual behaviour than a single-item measure can do.

## Conclusion

It seems that this study was successful in countering a trend towards unsafe sex within steady relationships, a difficult behavioural goal to achieve in HIV prevention. Although, in this study, the counselling protocol was used solely for MSM, it could easily be adapted and used for other risk groups such as heterosexuals with high-risk behaviour. Its principles of enhancing people's ability to elicit behaviour change through a better ability to think and appraise their risk behaviour can also be relevant to other people with HIV risk behaviour. Such counselling can easily be implemented in the routine setting of a health service, and carried out by public health nurses. The counselling was very well received by the participants, and the 15-minute counselling session was easy to implement within the vaccination appointment that was carried out by public-health nurses.

## Competing interests

The authors declare that they have no competing interests.

## Authors' contributions

MW carried out the study, participated in the design of the study, performed the analyses and drafted the manuscript. JdW, HH, JHR participated in the design of the study, discussed interpretation of results and helped to draft the manuscript. OdZ conceived of the study and participated in its design, and helped to draft the manuscript. All authors read and approved of the final manuscript.

## Pre-publication history

The pre-publication history for this paper can be accessed here:



## Supplementary Material

Additional file 1 **test results three-way ANCOVA, for testing intervention effects on UAI with steady and casual partners combined, with steady partners, and with casual partners**. The data provided represents the test results for intervention effects on UAI with steady and casual partners combined, with steady partners, and with casual partners. Click here for file
