# Supplementary material for: Effects of a short individually tailored counselling session for HIV prevention in gay and bisexual men receiving Hepatitis B vaccination
Source: BMC Public Health. 2009 Jul 21;9:255. doi: 10.1186/1471-2458-9-255 (PMC2726145; doi:10.1186/1471-2458-9-255)
Supplement: Additional file 1 — test results three-way ANCOVA, for testing intervention effects on UAI with steady and casual partners combined, with steady partners, and with casual partners. The data provided represents the test results for intervention effects on UAI with steady and casual partners combined, with steady partners, and with casual partners. [file 1471-2458-9-255-S1.doc]

Additional file 1: test results three-way ANCOVA, for testing intervention effects on UAI with steady and casual partners combined, with steady partners, and with casual partners

|  | UAI with casual and steady partners^1^ | | | | UAI with steady partners^2^ | | | | UAI with casual partners^3^ | | | |
| --- | --- | --- | --- | --- | --- | --- | --- | --- | --- | --- | --- | --- |
|  | Model 1 | | Model 2 | | Model 1 | | Model 2 | | Model 1 | | Model 2 | |
|  | F | p | F | P | F | p | F | P | F | p | F | P |
|  |  |  |  |  |  |  |  |  |  |  |  |  |
| Study condition | 12.58 | <0.001 | 11.57 | <0.01 | 4.96 | <0.05 | 10.37 | <0.01 | 16.57 | <0.001 | 0.36 | 0.55 |
| Pre-test UAI score |  |  | 42.72 | <0.001 |  |  | 48.08 | <0.001 |  |  | 236.54 | <0.001 |
| Steady relationship change | 6.12 | <0.05 | 8.89 | <0.01 | 0.08 | 0.79 | 6.77 | <0.05 |  |  |  |  |
| Pre-test sexual risk behaviour | 20.27 | <0.001 | 0.05 | 0.83 | 50.38 | <0.001 | 0.08 | 0.78 | 26.44 | <0.001 | 5.50 | <0.05 |
| Study condition by  Steady relationship change | 3.59 | 0.06 | 4.00 | <0.05 | 4.19 | <0.05 | 8.22 | <0.01 |  |  |  |  |
| Study condition by  Pre-test sexual risk behaviour | 2.69 | 0.10 | 1.75 | 0.19 | 0.23 | 0.63 | 0.07 | 0.79 | 17.76 | <0.001 | 0.72 | 0.40 |

^1^ Df_M_ = 1, 254

^2^ Df_M_ = 1, 255

^3^ Df_M_ = 1, 270

Note: Model 1: covariates include ethnic background (Dutch vs non-Dutch) and educational level (low level: elementary school, vocational school or lower-level high school vs high level: university, college, or higher- level high school)

Model 2: As model 1, with pre-test UAI- score as additional covariate
